# Recommendations From the Twitter Hashtag #DoctorsAreDickheads: Qualitative Analysis

**DOI:** 10.2196/17595

**Published:** 2020-10-28

**Authors:** Anjana Estelle Sharma, Ziva Mann, Roy Cherian, Jan Bing Del Rosario, Janine Yang, Urmimala Sarkar

**Affiliations:** 1 Department of Family & Community Medicine University of California San Francisco San Francisco, CA United States; 2 Center for Vulnerable Populations University of California San Francisco San Francisco, CA United States; 3 Ziva Mann Consulting Newton, MA United States; 4 Department of Culture and Theory School of Humanities University of California, Irvine Irvine, CA United States; 5 Berkeley School of Public Health University of California Berkeley Berkeley, CA United States; 6 Drexel University College of Medicine Philadelphia, PA United States

**Keywords:** social media, patient engagement, Twitter messaging, missed diagnosis, internet, physician patient relationship

## Abstract

**Background:**

The social media site Twitter has 145 million daily active users worldwide and has become a popular forum for users to communicate their health care concerns and experiences as patients. In the fall of 2018, a hashtag titled #DoctorsAreDickheads emerged, with almost 40,000 posts calling attention to health care experiences.

**Objective:**

This study aims to identify common health care conditions and conceptual themes represented within the phenomenon of this viral Twitter hashtag.

**Methods:**

We analyzed a random sample of 5.67% (500/8818) available tweets for qualitative analysis between October 15 and December 31, 2018, when the hashtag was the most active. Team coders reviewed the same 20.0% (100/500) tweets and the remainder individually. We abstracted the user’s health care role and clinical conditions from the tweet and user profile, and used phenomenological content analysis to identify prevalent conceptual themes through sequential open coding, memoing, and discussion of concepts until an agreement was reached.

**Results:**

Our final sample comprised 491 tweets and unique Twitter users. Of this sample, 50.5% (248/491) were from patients or patient advocates, 9.6% (47/491) from health care professionals, 4.3% (21/491) from caregivers, 3.7% (18/491) from academics or researchers, 1.0% (5/491) from journalists or media, and 31.6% (155/491) from non–health care individuals or other. The most commonly mentioned clinical conditions were chronic pain, mental health, and musculoskeletal conditions (mainly Ehlers-Danlos syndrome). We identified 3 major themes: disbelief in patients’ experience and knowledge that contributes to medical errors and harm, the power inequity between patients and providers, and metacommentary on the meaning and impact of the #DoctorsAreDickheads hashtag.

**Conclusions:**

People publicly disclose personal and often troubling health care experiences on Twitter. This adds new accountability for the patient-provider interaction, highlights how harmful communication affects diagnostic safety, and shapes the public’s viewpoint of how clinicians behave. Hashtags such as this offer valuable opportunities to learn from patient experiences. Recommendations include developing best practices for providers to improve communication, supporting patients through challenging diagnoses, and promoting patient engagement.

## Introduction

Twitter is a social media platform for users to share 280-character posts. Globally, Twitter included an average of 145 million daily active users in 2019; [1] 22% of Americans use the platform [2]. Twitter serves as an appealing resource to 61% of American adults who look for web-based health information [3], of which 12% use Twitter or social networking to share health updates [4-6]. Patients and families can develop communities for specific medical conditions and health care–related education [7,8]. Health care professionals utilize Twitter for networking, disseminating medical information, policy and research, disease and health communication monitoring, and advocacy for specific issues [9-11]. By providing this open forum, Twitter enables novel forms of dialogue among and across patients, patient advocacy groups, and health care professionals.

Twitter *hashtags* are words or phrases that Twitter users include in their posts to demarcate posts by a common theme or content. The hashtag can be entered as a search term and demarcate a particular dialogue on the website; therefore, it is a method of indexing conversations. In October 2018, a hashtag titled #DoctorsAreDickheads emerged on Twitter. The term originated from a professional YouTube video maker, who posted a video on Twitter explaining that she had been diagnosed with Ehlers-Danlos syndrome and postural orthostatic tachycardia syndrome (POTS) [12]. After describing her 8-year-long process with various health care professionals to receive the diagnosis, she closed the video with the phrase, “doctors are dickheads.” As users began to respond, #DoctorsAreDickheads emerged as a viral hashtag; patients, caregivers, and health professionals responded to the phrase by sharing their own experiences and criticizing the use of the hashtag. As of July 2020, the hashtag had been used in a total of 37,624 tweets by 12,731 Twitter user accounts and is still in active use (personal communication with Symplur, August 7, 2020).

We sought to describe the concepts represented in this hashtag as part of a broader depiction of patient-driven communications about health. To this end, we conducted a qualitative, phenomenological analysis of this hashtag. Our goal was to describe the *who*, the *what*, and the *how* of this phenomenon: who is posting the hashtag (as per their health care–related role), what is being stated with this hashtag or what are the common medical conditions associated with the hashtag, and how dialogue and prevalent concepts related to it arose. These concepts highlight specific patient and clinician challenges that are voiced publicly on social media and will inform future efforts to improve the patient’s experience of care.

## Methods

### Data Collection

We reviewed all tweets with the hashtag using a report generated from Symplur, a health care social media analytics company [13]. The greatest peak of this hashtag was in late October 2018 (see Multimedia Appendix 1 for the frequency of hashtags over time).

We then obtained tweets containing the hashtag dated between October 24 and December 31, 2018, comprising the predominant wave of use of the hashtag. We were able to obtain all tweets in this period, except for October 25, 2018, because of Symplur’s export limit of 2000 tweets at a time. In total, there were 9670 original tweets during the selected period, of which we were able to extract 8818. We randomly sampled 5.67% (500/8818) tweets for analysis using a random number generator. We selected 500 as the sample size based on a precedent social media analysis; Chan et al [14] demonstrated that a qualitative analysis of a sample of 540 tweets was sufficient to identify themes in how individuals understand and engage in health behaviors. We downloaded all tweets into Google Sheets spreadsheets (Google) for data abstraction and coding and used Microsoft Excel (Microsoft) for tabulation and thematic analysis.

### Data Processing of User Characteristics

We excluded tweets from abstraction if they were not in English, were nonsensical in content, or clearly posted by a bot (eg, had *bot* in the Twitter user profile). As Symplur exported the link to the original tweet, coders in the team reviewed the original Twitter post on the web and read the first tweet above or below the tweet of interest for context when necessary, as tweets can be either standalone comments or part of a conversation or *thread*. Team members abstracted the demographic role of Twitter users by reviewing the user profile and content of the tweet. For example, if a user described an experience while receiving medical care in their tweet, we abstracted their role as *patient*. If a user described themselves as an advocate for a clinical issue in their Twitter profile, we classified them as *advocate*. As there was a substantial overlap in role between *patient* and *patient advocat*e, as evidenced by tweet content or profile, we developed a shared category called *patient or patient advocate*. The team also abstracted the clinical conditions mentioned in the tweets, for example, *depression* or *fibromyalgia*.

### Analysis of Themes

This was an interpretative phenomenological analysis, in which a *phenomenon* or lived experience is described by exploring the perspectives and shared meaning of those who have experienced it [15]. We sought to understand for whom, for what, and how the *viral* hashtag #DoctorsAreDickheads became a way to frame and represent patients’ lived experiences on Twitter. We used the iterative process known as a hermeneutic cycle, moving continuously between data collection, interpretation, and theorization and incorporating awareness of our subjective perspectives as researchers in order to develop a nuanced analysis of the phenomenon [16]. This helped us understand how patients interpolate their individual experiences into a wider discourse of patient experience through the hashtag #DoctorsAreDickheads.

Team members (AS, ZM, RC, JY, and JD) coded 20.0% (100/500) sampled tweets together and the remainder individually. Each team member first independently reviewed the content of 50 tweets, selecting a short *code* of a word or short phrase describing the conceptual topics represented in tweets with the hashtag #DoctorsAreDickheads. These codes ascribed what phenomenologically could be called the *essence* or the core meaning of a tweet. Afterward, the team compared codes into a preliminary codebook. The team then reapplied the preliminary codebook to the 50 previously coded tweets and then used it to code another 50 tweets independently. The team then met once more to review code application, discuss new codes, and begin the process of interpretation. Two research team members reconciled differences in code application for the initial 100 tweets that were coded by the entire research team. Once consensus and agreement had been established, we divided the remaining 400 posts among 5 members of the team and coded them independently using the codebook and developing new codes when necessary.

Throughout the process, team members wrote memos or notes to describe shared meaning from the individual codes. After all remaining tweets were coded, the team sorted and categorized memos based on thematic content, developing an interpretive framework of the hashtag #DoctorsAreDickheads to result in final themes. When discussing possible themes, we also conducted frequency checks to prioritize the themes that were most frequently identified in the data. We aimed to identify all *meanings* of the phenomenon included in the sample; thematic saturation is not a priority in a phenomenological approach [17].

This study was reviewed by the University of San Francisco, California, institutional review board and categorized as *exempt*, with the approval number 19-27965.

## Results

### User Characteristics

Of the 500 tweets analyzed in our sample, 9 were excluded. Reasons for exclusion included being in a language other than English (n=2), written by a bot owing to having *bot* in the profile or nonsensical content (n=5), or containing no content (n=2). The sample included 344 independent Twitter users, with a median tweet frequency of 1 per user; 1 account had 7 posts, 1 had 20 posts, and 1 had 22 posts. In total, 50.5% (248/491) of tweets were posted by patients or patient advocates. Almost one-third or 31.6% (155/491) of tweets were posted by people in the other or unknown category. Health care professionals contributed 9.6% (47/491) of tweets; 4.3% (21/491) tweets were posted by caregivers, 3.7% (18/491) were posted by academics or researchers, and 1.0% (5/491) were posted by media or non–health care organizations. A list of roles identified in the coded sample is available in Table 1.

**Table 1:** Characteristics of Twitter users posting the hashtag #DoctorsAreDickheads (N=491).

Demographics represented in the sample^a^	Values, n (%)
Patient and/or patient advocate	248 (50.5)
Health care provider	47 (9.6)
Caregiver and/or family member	21 (4.3)
Researcher or academic	18 (3.7)
Media, non–health care organization	5 (1.0)
Non–health care individuals or unknown or other	155 (31.6)

^a^Some Twitter posts pertained to multiple demographics.

In our sample, 60.2% (296/491) tweets mentioned a clinical condition. The most common condition mentioned was chronic pain (44 tweets). Mental health, musculoskeletal, and obstetrical or gynecologic conditions and procedures were also common. Ehlers-Danlos syndrome was the most common specific condition, followed by fibromyalgia, chronic fatigue syndrome (also known as myalgic encephalitis), POTS, and mast cell activation syndrome. A full list of conditions is available in Table 2.

**Table 2:** Clinical conditions mentioned in sample tweets (n=296).

Condition	Number of tweets
Chronic pain	44 (general pain: 38; fibromyalgia: 5)
Mental health	31
Musculoskeletal conditions	26 (Ehlers-Danlos syndrome: 19; other: 7)
Obstetrical or gynecological conditions or procedures	21
Neurological conditions	18 (chronic fatigue syndrome or myalgic encephalitis: 5; POTS^a^: 4; other conditions: 9)
Disability	17
Chronic illness (unspecified condition)	14
Gastrointestinal conditions	8
Autoimmune conditions	7 (mast cell activation syndrome: 4; other autoimmune conditions: 3)

^a^POTS: postural orthostatic tachycardia syndrome.

### Major Themes

We identified 3 core thematic results that were manifested in the experiences represented within our sample. Full definitions of each theme and additional exemplar quotes are found in Table 3. Of note, we are publishing verbatim tweets with usernames to give credit to Twitter users and their contributions to this discourse when possible. We obtained permission from cited users to publish these tweets. For tweets about which we received no response, we anonymized the content in accordance with recommendations regarding social media research [18].

**Table 3:** Major themes and definitions identified in the content analysis of the Twitter hashtag.

Theme	Definition	Example tweets
Belief and diagnosis	Describing experiences with medical providers being skeptical, dismissive, or “gaslighting”; this disbelief then causing delayed or incorrect diagnosis and/or medical harm	“It took 10 years for my MS to be diagnosed. Doctors thought I was embellishing my symptoms and doing too much internet research. If they had spent that time listening, running the correct tests, and treating me, I might not be disabled to the point I am now. #DoctorsAreDickheads” (@VenusDoom14) “Two cardiologists dismissed my POTS as ‘nothing wrong’ or ‘it’s all in your head’ before the third one figured out my POTS. He’s a lifesaver, but the other #DoctorsAreDickheads” (@Snarcoleptic_13) “this stings so hard when #DoctorsAreDickheads do this to you while gaslighting you about the psychosomatic nature of your symptoms” (@moniquedhooghe) “I went to an urgent care for what turned out to be pneumonia but had to spend half of the appointment being grilled over why I ‘think’ I have epilepsy. ‘Because the neurologist I've been seeing for a decade told me,’ was not good enough. #DoctorsAreDickheads” (@Jenny_Trout) “One doctor I went to, without even knowing me or my history, interrupted me while I was explaining my symptoms & just said ‘You have a psychological condition.’ I said no I don’t.. & he cut me off again & said ‘Yes you do.’ #DoctorsAreDickheads (@d_vaz)
Power inequity in the patient-provider interaction	Differential in power (due to medical hierarchy as well as misogyny, White supremacy, and ableism) affecting communication and behaviors between clinicians and patients	“All I want is to be believed. To have people understand that when sick/stressed, I can't pretend or act and so my intonation is flat. But they won't. And, if I wasn't hairy, if I didn't have external ‘plumbing,’ this would be worse. #DoctorsAreDickheads” (@theAutistech) “Well, the time to care about my well-being is when I’m in the clinic, but physicians often will not. More often if they are men, and particularly more often if they’re white. This isn’t a stereotype, it’s established in the research. #DoctorsAreDickheads” “#DoctorsAreDickheads is being driven by people living with disabilities and activists that I know. I feel this so deeply – I’ve experienced this bullsh*t even if I’m closer to neurotypical – they confuse us and ignore our own knowledge about our bodies.” “Physicians have all the power. They could help us get better, but for all of us with chronic illness, they’ve traumatized us. We’re too scared to come in to be seen. You can’t get it unless you’ve lived it. #DoctorsAreDickheads”
Metacommentary	Discussion about the rationale for and impact of this hashtag in public discourse	“To all the people that are using #DoctorsAreDickheads first off all Get stuffed (*insert: crying laughing emoji*) our grouping all doctors into a group that in reality is only made up of like 1% of them. Now am I saying that all doctors are amazing? No but a lot of them work f**king hard and spend time helping others when they could be at home with there family” (@PineappleYT123) “People complaining about the #DoctorsAreDickheads hashtag because it contains a vulgarity... Do you know if patients use curse words (what I call “cuss words” from home) in a medical practice, they can be labeled ‘difficult’?” (@DrZackaryBerger) “I'm sorry, but #DoctorsAreDickheads is simply honest. Some doctors are rude, some are abusive, some are incompetent. Some are brilliant, but that doesn't mean we can't discuss the generally poor response to patients who raise issues.” (@WTBDavidG) “I've seen as many if not more medical professionals responding positively to #DoctorsAreDickheads in 24hr than I have to more polite debate in the last 8 months.” (@stendec6) “Decent doctors knows they are decent. They understand why the hashtag exists and why patients are suffering. They can deal with a few hurt feelings because they see the greater change that is possible when we stand up for ourselves #DoctorsAreDickheads” (@IntactCervix) “So much trauma is due to us doctors. We learn best from our patients, but these lessons come too late. The stigma about weight isn’t something we talk about in our training. Let’s do better. #DoctorsAreDickheads”

### Belief and Diagnosis

Patients and caregivers described a common experience of clinicians not listening, not believing, minimizing, or not valuing their accounts of illness. The experience of being disbelieved was often linked to experiencing an incorrect, delayed, or missed diagnosis. These diagnostic adverse events were associated with physical harm:

Had terrible blood clots for several years–they said my legs were hurting from fibro and they couldn’t do anything. Then I started having trouble breathing and we dashed to the ER. The clots had ended up spreading from my legs to my lungs.

In addition to physical harm, others described emotional harm and guilt:

I felt horrible. I was ruining all the holidays, and I could not do a single thing about it. I felt like a worthless piece of crap. And all because... the doctors didn't look at me, did not see my pain as valid. Even now, with my diagnosis.. it is hard… #DoctorsAreDickheads.

@WheelieNick

The narratives with this theme described prevalent gaslighting, meaning a manipulative tactic in which someone questions a person’s perceptions, memories, and sense of reality. They also described egotistical behavior from clinicians, lording medical training or expertise over patients or being dismissive of patient input when their diagnosis or assessment was challenged:

A doc told me that I had a cancer syndrome. I said no way – I had been in an accident right before my symptoms started. He told me, “I have an Ivy League degree, so don’t ask questions.” Turns out he was incorrect. No cancer. #DoctorsAreDickheads.

Within this theme, we observed how patients countered the narrative of being dismissed by using #DoctorsAreDickheads to create a community where people are believed. In response to a thread in which a patient shared how they “sobbed . . . heaved with the realization that yet again (I’m) being gaslighted about (my) own damn body,” another Twitter user responded:

...please, consider the #DoctorsAreDickheads conversation. This hashtag shows that you're not on your own in this. It isn’t just you imagining things. Look at all the people here who believe your words.

### Power Inequity in the Patient-Provider Interaction

Twitter users described their experiences using this hashtag as a result of the power inequity and hierarchy in medical care. Clinicians hold power in decision making and medical orders, serving as *gatekeeper* for desired services. This included experiences where clinicians denied patient bodily autonomy:

And then after I was finished having kids and wanted my tubes tied, the first two doctors I asked, refused. For non evidence based reasons. It took me 2.5 years to find a doctor who would. #DoctorsAreDickheads.

@MxPeachyKi

The power imbalance impacts communication, and a number of patients described being aware of what they felt they could or could not say, or *self-edit* what they would express in the visit, to protect themselves from consequences that would affect their care:

Honestly, if we suddenly go very silent and compliant, we're actually fighting back rage and tears bc we know damn well if we let it show you'll just label us hysterical and FIRE us as patients. Like we somehow serve YOU. #DoctorsAreDickheads.

@rhysfelis

Many of the accounts recounted experiences where patients felt that the clinicians were abusing their power. These experiences ranged from subtler, verbal diminishments of the patient experience to physical, verbal, and even sexual abuse and/or severely unethical care:

cw: sexual assault I had many doctors actively try to cover up or push under the rug the fact that their coworker sexually assaulted me when I was 14. All these people are still practicing at a major hospital, including assaulter. Suffice to say those #DoctorsAreDickheads.

@atoradegay

Within this theme was a call for attention to how the patient or clinician power differential is compounded by structural inequities in society. Tweets addressed how White, cisgender, and neurotypical patients have more privileges in medical visits because of structural power imbalances. People of color, lesbian, gay, bisexual, transgender, queer individuals, and people living with disabilities described an intersectional experience, shaped from their identities as members of marginalized groups, in which there was a higher risk of a negative encounter or inappropriate care:

My #DoctorsAreDickheads story: before I’m a physician, I’m a queer woman. Physicians, nurses, and everyone in healthcare have a long systemic history of abuse of power and broken trust with the LGBTQ+ community. My family & my community fear medicine because of it. Don’t @ me.

@ShannonOMac

THANK YOU. It was a little frustrating bc most of the participants were white and I didn't quite know how to articulate that #DoctorsAreDickheads is different when you have other marginalized IDs outside of being disabled or sick.

@Twitchyspoonie

### Metacommentary on Hashtag

Although much of the conversation using this hashtag focused on narratives of experiences with clinicians, there was also a *meta* conversation about the meaning of the hashtag’s use on Twitter. This included the risks and benefits of using such an inflammatory term:

Yes this #DoctorsAreDickheads represents poor experiences of care. We try to and should improve this if needed.But doctors are humans, patients, parents, and professionals. Attacking us is counterintuitive when we campaign for improvements. #twitter is this really helpful?

@dr_nigel_lane

Patient advocates noted that the hashtag got attention, precisely because it was sensational:

The provocative hashtag #DoctorsAreDickheads drew people's attention to widespread, systemic medical maltreatment. A more polite hashtag couldn't have done this.

@jeff_says_that

Patients, advocates, and health care providers shared their frustration and hoped that this attention and dialogue would allow better understanding and possibly change:

The medical professionals getting butthurt by #doctorsaredickheads need to read it for what it is - our cries to be treated as PEOPLE first, CONDITIONS second. A desire for inclusivity and a genuine desire to help IMPROVE patient care and the doctor-patient relationship.

@Chrisa_Hickey

Within the metacommentary, a parallel hashtag emerged, *#DoctorIRespect*, used to share accounts of laudable or appropriate medical care. These tweets contained recommendations for improved communication during medical visits. Some tweets contained suggestions for engaging patients as partners in their care and system redesign to ensure more patient-centered care:

So how do you become a #DoctorIRespect? It’s really easy. If you have no clue what is going on, just say so. Tell us you don’t know. That’s all.

If you’re not involving patients, I urge you to begin doing so. Heck, there are many patients that have experience in orchestrating such change within large medical spaces (insert: waving hand emoji)We’re here with experience and even degrees. Hire us to help you. #DoctorsAreDickheads

@GraysonGoal

## Discussion

Our study paints a picture of patients living with chronic conditions, lacking power within the medical encounter, and turning to social media to share testimonies of being disbelieved and disrespected. Our study is the first empirical approach to analyze the phenomenon of this hashtag on Twitter, utilizing a random sample. Another response was an editorial piece, which concluded that the degree of rancor in the conversation would not aid in improving medical care [19]. Rare or challenging clinical syndromes were commonly mentioned, such as Ehlers-Danlos syndrome, chronic pain, and chronic fatigue syndrome, which have been seen in other analyses of medical topics discussed on Twitter [20,21]. Twitter may be an underutilized resource for understanding the patient’s perspective and provider dynamics within the diagnostic pathway, particularly for challenging-to-diagnose conditions; social media data can be mined to monitor care quality and patient experience [22].

The connection between communication within the clinical encounter and the ability to make a correct and timely diagnosis was an unexpected finding. The hashtag #DoctorsAreDickheads highlighted how clinician engagement with patients is not just a matter of patient experience, but a priority for diagnostic safety. Patients shared how disrespectful treatment was connected to a missed, delayed, or incorrect diagnosis. Several previous studies on Twitter data found descriptions of procedural, medication, and diagnostic errors [17,23]; our larger sample validates that patients can self-identify and report diagnostic adverse events. How information is conveyed or received is highly dependent on belief and respect throughout the encounter. Improvements are urgently needed in how clinicians communicate and how patients are involved in the diagnostic pathway [24,25].

The central takeaway from our analysis was how patients felt disempowered, disrespected, and disbelieved. Patients described a range of ways in which they are vulnerable during a health care encounter [26]. Beginning with the existing power differential between provider and patient, tweets described how patients enter the health care system struggling with active symptoms of illness or health needs and can be further burdened by bias, including racism, misogyny, or ableism in the medical system, or by previous traumatic experiences with health care. These vulnerabilities could lead to avoidance or *self-editing*, which hashtag users described as trying to behave like a *good patient* rather than continuing to share their knowledge or expertise in their condition. When experiencing mistreatment, patients are not engaged in sharing knowledge and expertise in their conditions, further reducing the quality of the clinical encounter. Our findings resonate with other works detailing the impacts of power and hierarchy in medicine, in which the lack of agency or respect further perpetuates harm [27,28].

In addition to describing harm from misdiagnosis, patients described assaultive behavior from health care professionals, ranging from hurtful comments (eg, ableist or sexist remarks, carelessness about prognosis) to egregious, unethical, or illegal acts (including sexual abuse). Traditionally, the patient-clinician interaction is private, with few witnesses. Should there be abuse, patients do not have clear-cut routes of action. In the climate of #MeToo and the Black Lives Matter movement, people in positions of power are being held to greater accountability for their behavior. As this hashtag demonstrates, Twitter provides greater transparency to clinician behaviors in a public forum.

Using this hashtag, tweets demanded empathy on behalf of both clinicians and patients. Within the metacommentary theme, some hashtag users accused patients of exacerbating clinician burnout and mental illness. Patients and advocates responded by highlighting clinicians’ privilege, encouraging them to be less defensive and listening to critiques. This opposition between patient needs and clinician burnout is a false dichotomy and speaks to the deeper shortcomings of a medical system that erodes empathy both for patients and for health care personnel. As health care systems continue to explore changes to improve quality and patient experience, work to achieve the quadruple aim is aligned with improving both clinician workplace satisfaction and patient experience [29]. Both patient and clinician wellness are priorities; however, the #DoctorsAreDickheads conversation shows how power differentials and limitations in the current health care system disproportionately impact patients, putting their needs in conflict with providers.

**Figure 1:**
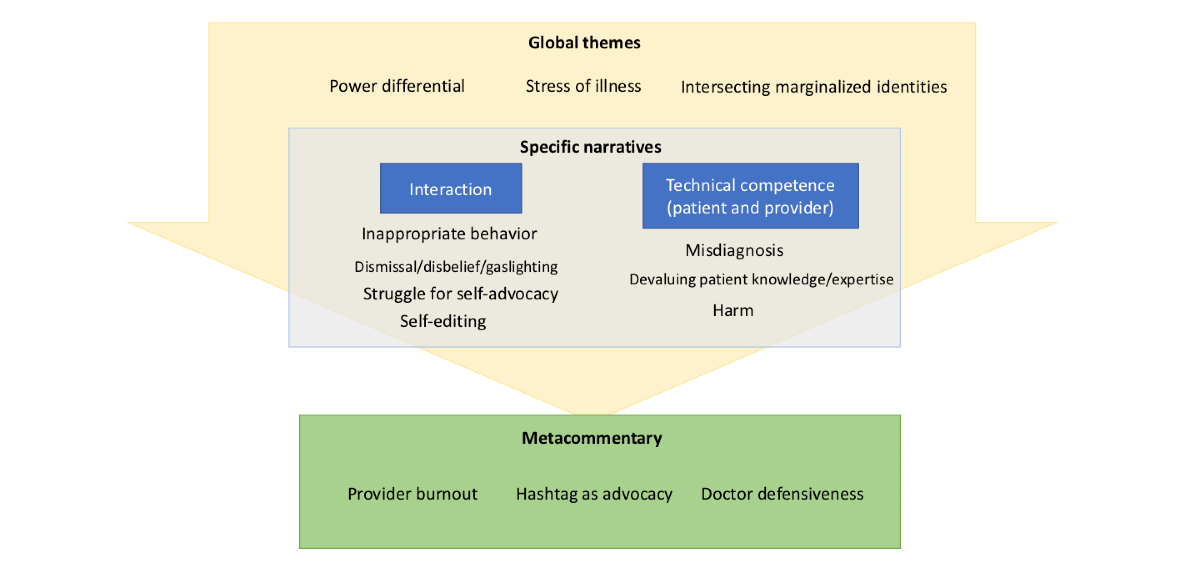
Map of major themes identified in Twitter hashtag.

Figure 1 shows how the themes relate to each other. The direct experiences of gaslighting, minimization, and ignoring patient expertise (Theme 2) are couched in the overarching themes of power differential, the burden of illness, and historically marginalized identities (Theme 1). In metacommentary, clinicians have responded both defensively and in support, and patients and advocates noted the hashtag’s utility to gain attention for advocacy for systemic changes (Theme 3).

**Figure 2:**
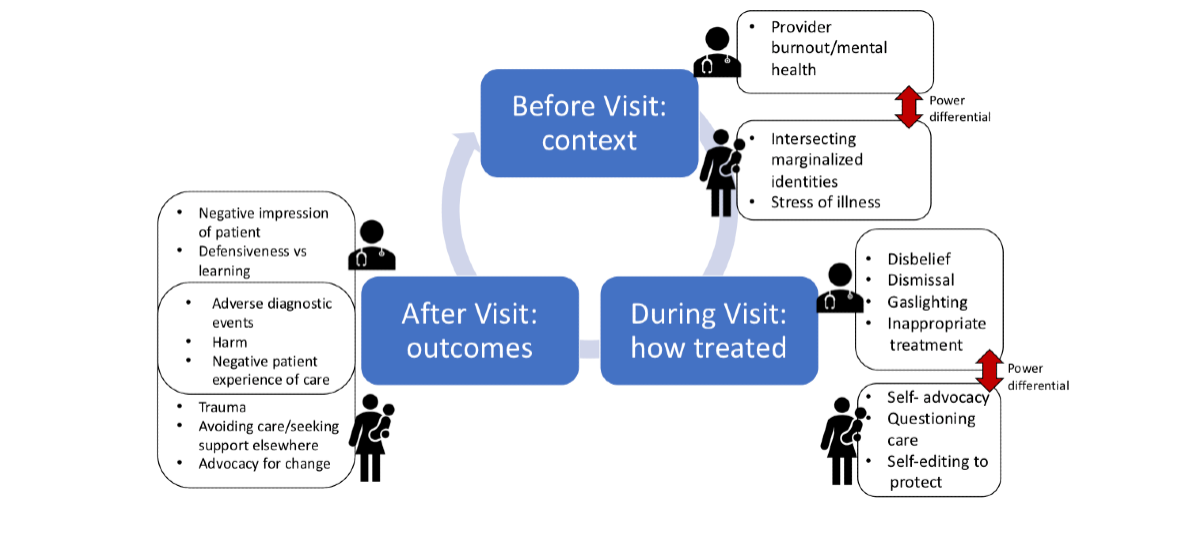
Cyclical process map of themes identified in Twitter hashtag.

We organized the experiences described in a process map of the patient and clinician encounter, showing how major themes function in a cyclical fashion (Figure 2). First, contextual factors, including the power differential, the patient’s intersectional identity, and provider well-being, affect the clinical interaction before a visit. During the encounter, the power differential affects the interaction in which the clinician may disbelieve, devalue, or *gaslight* the patient, who may engage in various coping behaviors such as self-advocacy, self-editing, or questioning care. In this interaction, downstream negative outcomes include misdiagnosis, medical harm, and negative patient experience or trauma. In this conceptual map, harmful outcomes are shared, highlighting the message of the #DoctorsAreDickheads hashtag as a call to action to improve outcomes for both patients and clinicians.

Actionable recommendations for clinicians were provided with this phenomenon. First, clinicians can strive to diminish their defensiveness when patients share a negative experience. Second, there is the potential to improve how clinicians communicate in a clinical encounter. Trainings may improve clinician capacity to listen about a health concern, voice diagnostic uncertainty, even by stating “I don’t know,” and accompany patients through challenging diagnoses or chronic conditions. Third, patients encouraged increased patient engagement within health care systems, such as through community advisory boards or hiring patients as consultants, to develop more patient-centered care systems [30].

### Limitations

Limitations of this study include a relatively small sample of events; however, our initial sample of 500 is similar to other qualitative analyses of social media posts [8,11]. Those who post on Twitter are not representative of all patients; however, given that 55.2 million people in the United States use Twitter, it is clear that Twitter’s users are a sizable proportion of enrolled patients [31]. We do not know if patients who are higher utilizers of health care are more or less likely to post content on Twitter using this hashtag. Finally, we do not know detailed demographic information about Twitter users or their geographic location.

### Conclusions

Twitter and social media are growing platforms where patients discuss health care; this public forum holds clinicians to a higher level of accountability and transparency. #DoctorsAreDickheads is an intentionally sensational hashtag, born out of frustration with health care interactions. The hashtag is meant to raise awareness of common negative patient experiences, particularly for those living with challenging, rare, and chronic conditions. Patients experience disbelief, mistrust, and lack of listening from their clinicians, which they link to delayed or missed diagnoses. Patients asked for a deeper recognition of the capacity and expertise they bring to the clinical visit and awareness of how power and bias affect the encounter. Although clinicians may feel resistant to concerns expressed through social media, patient advocates on Twitter advocate for system-level improvements to improve medical treatment and patient experience. By systematically exploring views expressed on these platforms, clinicians and health care leaders may identify important areas for improvement, such as improved communication during a challenging diagnosis.

## Appendix

Multimedia Appendix 1 Prevalence of hashtag #DoctorsAreDickheads over time.

## Abbreviations

NIH: National Institutes of Health
POTS: postural orthostatic tachycardia syndrome
