# Correction: Recommendations From the Twitter Hashtag #DoctorsAreDickheads: Qualitative Analysis

**DOI:** 10.2196/25511

**Published:** 2020-11-09

**Authors:** Anjana Estelle Sharma, Ziva Mann, Roy Cherian, Jan Bing Del Rosario, Janine Yang, Urmimala Sarkar

**Affiliations:** 1 Department of Family & Community Medicine University of California San Francisco San Francisco, CA United States; 2 Center for Vulnerable Populations University of California San Francisco San Francisco, CA United States; 3 Ziva Mann Consulting Newton, MA United States; 4 Department of Culture and Theory School of Humanities University of California, Irvine Irvine, CA United States; 5 Berkeley School of Public Health University of California Berkeley Berkeley, CA United States; 6 Drexel University College of Medicine Philadelphia, PA United States

In “Recommendations From the Twitter Hashtag #DoctorsAreDickheads: Qualitative Analysis (J Med Internet Res 2020;22(10):e17595), the authors noted three errors.

In the originally published manuscript, the fourth sentence of the second paragraph of the Introduction section read:

The term originated from a professional YouTube video maker, who posted a video on Twitter explaining that she had been diagnosed with Ehlers-Danlos syndrome and postural orthostatic hypotension syndrome (POTS).

This has been changed to:

The term originated from a professional YouTube video maker, who posted a video on Twitter explaining that she had been diagnosed with Ehlers-Danlos syndrome and postural orthostatic tachycardia syndrome (POTS).

In Table 2, footnote ^a^ originally read:

POTS: postural orthostatic hypotension syndrome.

This has been changed to:

POTS: postural orthostatic tachycardia syndrome.

The Abbreviations section originally included the following:

**POTS:** postural orthostatic hypotension syndrome.

This has been changed to:

**POTS:** postural orthostatic tachycardia syndrome.

These corrections will appear in the online version of the paper on the JMIR Publications website on November 9, 2020, together with the publication of this correction notice. Because this was made after submission to PubMed, PubMed Central, and other full-text repositories, the corrected article has also been resubmitted to those repositories.

