# Supplementary figures and images for: Recommendations From the Twitter Hashtag #DoctorsAreDickheads: Qualitative Analysis
Source: J Med Internet Res. 2020 Oct 28;22(10):e17595. doi: 10.2196/17595 (PMC7652212; doi:10.2196/17595)

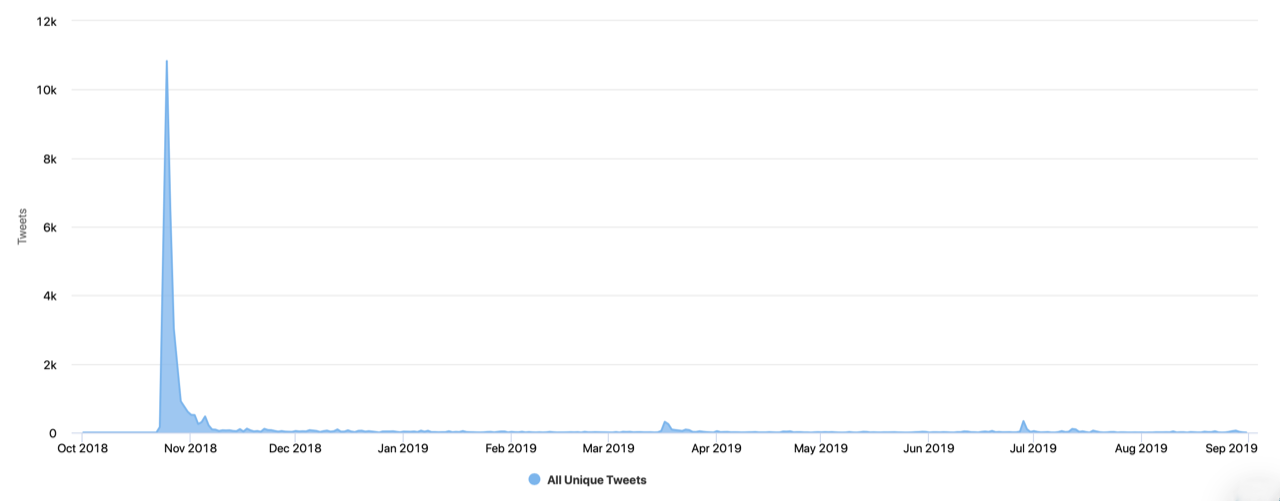

Supplement: Multimedia Appendix 1 [file jmir_v22i10e17595_app1.png]
